# Investigation of Polymer Biofilm Formation on Titanium-Based Anode Surface in Microbial Fuel Cells with Poplar Substrate

**DOI:** 10.3390/polym13111833

**Published:** 2021-06-01

**Authors:** Ahmet Erensoy, Nurettin Çek

**Affiliations:** 1 Department of Parasitology, Faculty of Medicine, Firat University, Elazig 23119, Turkey; aerensoy@firat.edu.tr; 2 Department of Metallurgical and Materials Engineering, Institute of Science, Mersin University, Mersin 33343, Turkey

**Keywords:** poplar biopolymer, microbial fuel cell, electron transfer, electricity

## Abstract

Microbial fuel cells (MFCs) have attracted attention by directly converting the bioelectrochemical energy possessed by the organic materials that make up the biomass into electrical energy. In this study, the relationship between the biofilm formed on the titanium-based anode electrode surface, and the chemical composition of the substrate, the energy source of MFC, was investigated. For this, MFCs were made by using poplar wood shavings rich in organic material as the substrate, titanium-based material as the anode electrode, and natural soil as bacterial habitat. Three types of MFCs containing 1%, 10%, and 20% poplar wood shavings by weight were made and named P1-MFC, P2-MFC, and P3-MFC, respectively. According to electrochemical analysis, P3-MFC provided the highest open circuit voltage with 490 mV value, and the highest power density with 5.11 mW/m^2^ value compared to other MFCs. According to optical microscopy examinations, there were *Bacillus* and *Coccus* species of bacteria in the soil structure, and these bacteria also existed around the fiber of poplar wood shavings in MFCs. Scanning electron microscopy (SEM), energy-dispersive spectrum (EDS), and Fourier transform infrared spectroscopy (FTIR) analysis showed that MFCs formed biofilm in the titanium-based anode, and the chemical composition of this biofilm with poplar tree was similar. As a result, due to the catalysis reactions of bacteria, the titanium-based anode electrode surface was coated with polymer biofilm released from poplar wood shavings.

## 1. Introduction

Microbial fuel cells (MFCs) directly convert the bioelectrochemical energy of organic structures in biomass into electrical energy through to catalysis reactions of bacteria. The basic structure of MFC includes the substrate, bacteria, and electrode [1,2]. Since the substrate and bacteria are in a liquid medium, their medium is at the same time the MFC electrolyte. Electrodes are electrically conductive materials. In MFC, the substrate expressed as the source of organic material is decomposed by bacteria, resulting in the release of electrons and protons. The electrons first go to the anode electrode and from there they reach the cathode electrode by using the outer circuit. Protons arrive at the cathode electrode using the electrolyte. Protons are combined with electrons and the oxygen supplied from the air to form water, at the cathode electrode. As a result of these processes, electrical energy is generated by the MFC [1,2,3,4].

The electrical energy generation of MFC also causes bacteria to form an organic biofilm layer, especially on the anode electrode surface [5]. The biofilm layer plays a very important role in the transfer of electrons to the anode electrode. In previous studies [5,6,7], it has been stated that the efficiency of the biofilm layer increases the electrical power density of MFC, and it has been reported that the production of extracellular polysaccharides by the bacteria leads to the formation of biofilms. In other words, at the stage of biofilm formation, bacteria secrete extracellular polymeric materials [7].

In another study [8], it was stated that the electrical performance of MFC is related to the physiology of anodic biofilm. In the same study, it was stated that the metabolic activity of the biofilm in the anode electrode dynamically responded to different substrates. This was in fact evidence that there is an interaction between the biofilm and its substrate structure, and that the interaction affects the electrical power density of the MFC.

More studies are needed on the properties of the biofilm layer formed on the electrode surfaces as a result of the reaction of rich polymer-containing substrate materials with bacteria and the effect of MFCs on electrical energy production performance. For this, poplar biomass can be used as a substrate in MFC, since there are various organic polymers such as cellulose, lignin, and polysaccharide used in the production of biopolymer in the structure of poplar wood shavings [2,9,10]. In a study [2], poplar wood shavings substrate was used for MFC, and graphite was used as the electrode; at the end of the experiments, the electrodes were examined by Fourier transform infrared spectroscopy (FTIR). According to FTIR results, polymer biofilms such as those containing polysaccharides and nucleic acid were detected on the graphite electrode surface [2]. The striking point here was that the cellulose material in the poplar wood structure was found in the biofilm structure on the anode electrode surface. In other words, cellulose, a natural polymer has been transferred from the substrate to the electrode biofilm. However, it is a matter of curiosity as to whether this is limited to the graphite electrode only, and whether it will happen with electrodes made from other materials.

In order to address this question, in this study, we produced MFCs by using titanium-based anode electrodes, graphite cathode electrodes, poplar shavings as substrate, and natural soil bacteria habitat. In these MFCs, only the substrate ratios are different from each other. Therefore, MFCs containing 1%, 10%, and 20% by weight of poplar wood shavings were called P1-MFC, P2-MFC, and P3-MFC, respectively. The electrochemical performance of these MFCs and the organic structure of the biofilms formed on the titanium-based anode electrode surface were examined and explained. 

## 2. Materials and Methods

### 2.1. Materials

Titanium-based electrodes (8 cm × 3 cm × 0.5 cm = 12 cm^2^) and graphite rod electrodes (0.75 cm diameter, 8 cm length) were procured from Toreci Machinery Trade, Istanbul, Turkey. Natural soil was obtained from Elazig, Turkey. BRUKER (Billerica, MA, United States of America) brand devices were used for scanning electron microscopy, energy-dispersive spectrometer, and Fourier transform infrared spectroscopy works. Optical microscope (SOIF BK5000-TR/L), digital multimeter (UNI-T UT 61C), and external resistance (Yuetai Group) were purchased from Shanghai, China.

### 2.2. Methods

Titanium (Ti)-based electrodes, which are referred to as biomaterials due to their excellent biocompatibility, bio stability, and bio affinity properties, were used as anode electrodes [11]. Graphite cylinder rod was used as the cathode electrode because it is biocompatible, inert, and does not interfere with bacterial growth [1]. Natural soil is a microbial environment where bacteria live; when is watery, it is a suitable material for the ionic conductor, mediator, separator, and bacteria to release electrons and protons from organic material [1,2,12,13]. Therefore, in this study, natural soil obtained from Elazig, Turkey was used as a microorganism habitat for MFCs. Bacterial analyses of this natural soil have been carried out in a previous study, and there was a mixed culture medium of *Enterococcus faecium*, *Bacillus thuringiensis*, and *Bacillus cereus* bacteria [2]. The use of this natural soil with the known presence and properties of bacteria was especially preferred in order to provide us with supportive information about biochemical processes. No chemical pretreatment or any chemical material treatment were carried out, and thus the natural environment, structures, and specific metabolic activities of bacteria in the natural soil would not be impaired. In this study, poplar wood shavings were preferred as the substrate for MFCs because they contain biopolymers such as cellulose, polysaccharide, and organic materials such as sugar and glycoside, and also the previous study reported that it is a suitable substrate for bacteria [2]. For this, poplar shavings that emerged during the cutting of trees were dried under sunlight for 10 days in the open air. Organic material structure of poplar wood shavings was examined by FTIR. Natural tap water was used to sustain bacterial life and ensure ionic conductivity in MFCs. The tap water used in the previous study was used [2]. Membranes were not used in MCF systems to keep internal resistance as low as possible, and single-chamber MFCs were manufactured [2]. The reactors of MFCs are plastic and have a volume of 100 milliliters (mL). For the setup of the MFCs, firstly, 50 g of natural soil was placed in plastic boxes with the same properties; then, its poplar wood shavings were placed up to 1%, 10%, and 20% of the soil weight. After this, titanium-based anode electrode and graphite cathode electrode were placed into all MFCs. MFCs containing 1%, 10%, and 20% by weight of poplar wood shavings were named P1-MFC, P2-MFC, and P3-MFC, respectively. By putting 40 mL of natural tap water into all MFCs, the metabolic activities of bacteria and the ionic transmission process of MFCs were initiated, that is, MFCs were operated. At the beginning and the end of the experiments, the chemical and morphological structure of the anode electrodes was examined by scanning electron microscopy, energy-dispersive spectrometer, and FTIR [2,11]. The bacterial structure of the electrolyte consisting of poplar wood shavings and soil was examined by optical microscope. In order to determine the electrochemical performances of the MFCs, we first operated on all MFCs under open-circuit conditions without any external load connected, and open circuit voltage (OCV) values were measured using a digital multimeter. When stable OCV values were provided, external resistances ranging from 10 to 221,000 ohms were connected to the MFCs, and the voltage across the resistance was measured with a multimeter. Each measurement on external resistances was made at 10-min intervals. Next, the measured voltage values in the resistances and the values of the resistances were applied to Equation (1) to detect the current values. After this, polarization curves were determined using the current and voltage values obtained from the resistances [2,12,13]. The current values of the MFCs were calculated using Equation (1).

(1) $$I=\frac{V}{R}$$

Here, *I* is the current, *V* is the voltage measured on the resistance, and *R* is the resistance value of the external resistances.

The power density of the MFCs was calculated by applying the values in the polarization curves and the anode electrode surface area value to Equation (2).

(2) $$P_{density}=\frac{I.V}{S}$$

Here, *P_density_* is power density, *I* is the current, *V* is the voltage measured on the resistance, and *S* is anode electrode geometrical surface area (m^2^).

## 3. Results

### 3.1. Electrochemical Performance of MFCs

**Figure 1:**
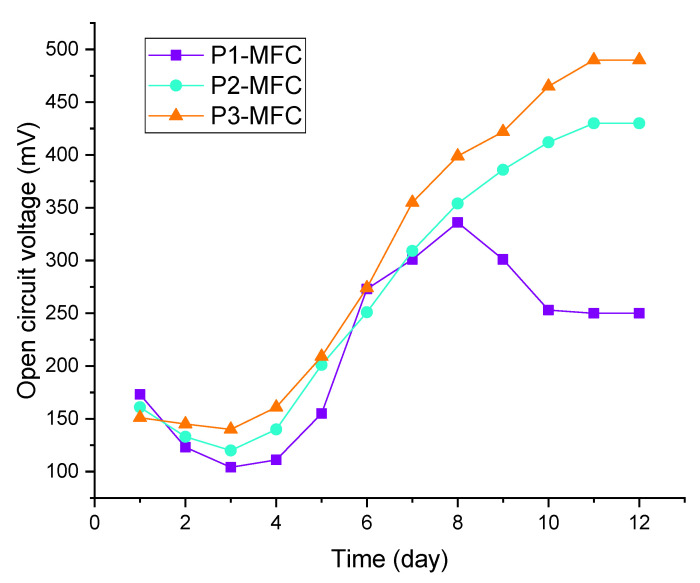
Daily measured OCV values of MFCs.

Open-circuit voltage (OCV) values of P1-MFC, P2-MFC, and P3-MFC varied from start to result. The highest OCV values of P1-MFC, P2-MFC, and P3-MFC were 336, 430, and 490 mV, respectively. These three MFC types reached stable OCV values after 11 days. The stable OCV values of P1-MFC, P2-MFC, and P3-MFC were 250, 430, and 490 mV, respectively. The daily OCV values of these MFCs are given in Figure 1.

**Figure 2:**
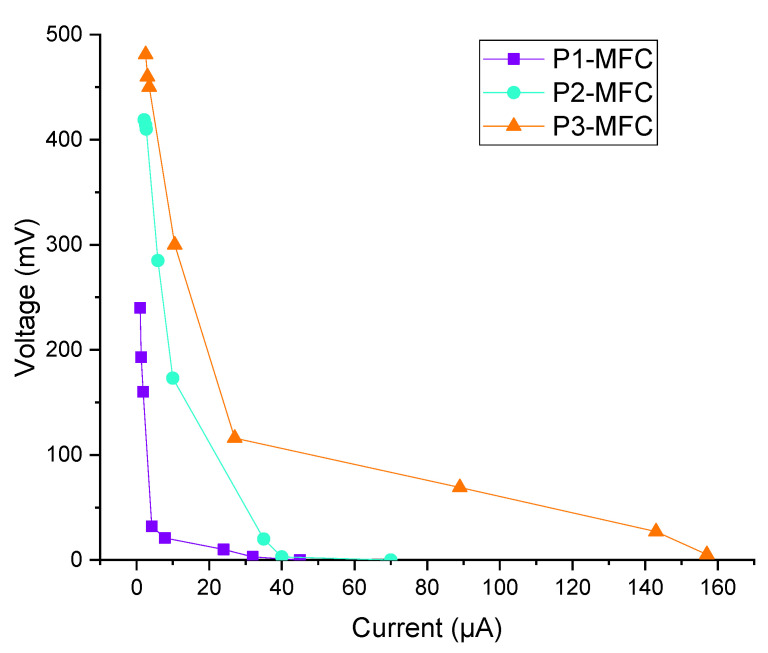
Polarization curves of MFCs.

The relationship between the voltage and current that occurs when the external resistance is connected to the MFC and the power and power density calculated as their function is determined by the polarization curve [3,14]. Polarization curves of P1 MFC, P2-MFC, and P3-MFC are given in Figure 2. As it can be seen in Figure 2, as the external resistance increased in all MFC, the voltage increased up to a certain level. However, this increase was not continuous, as the external resistance increased, the current value decreased.

**Figure 3:**
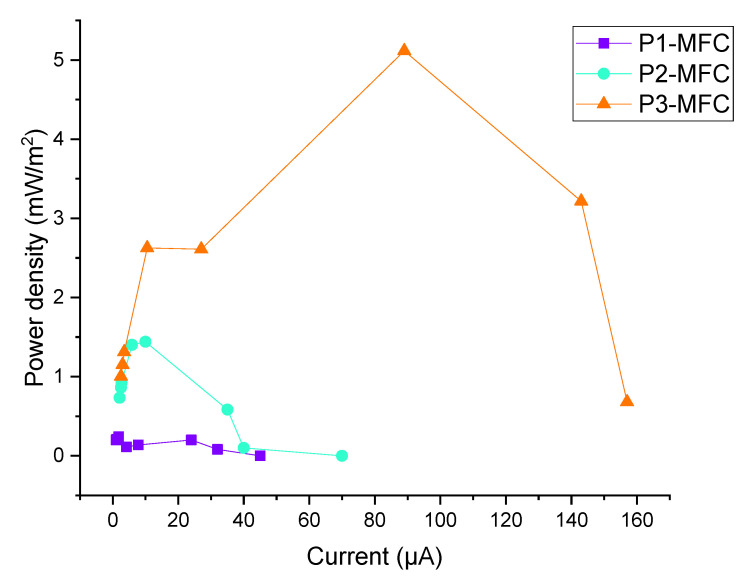
Power density curves of MFCs.

As can be seen in Figure 3, the highest power densities (peak point) of P1-MFC, P2-MFC, and P3-MFC were calculated as 0.24, 1.44, and 5.11 mW/m^2^, respectively. The power generation of MFCs indicates that poplar shavings serve as a suitable substrate. However, current values of MFCs varied. At low current, activation loss occurs first, followed by ohmic loss and loss of concentration. The high ohmic loss means that the MFC losses are mostly caused by electrodes rather than the substrate and microbial activity [15]. Furthermore, due to the fact that organic material can be catalyzed (oxidized) by bacteria, losses occur in the anode, which are expressed as overpotential losses. These losses include activation energy, microbial energy for maintenance and growth, ohmic losses, and concentration losses. Concentration losses are determined by the presence of substrate on the anode surface and the accumulation of products on the anode surface, substrate availability, substrate intake, and mass transfer [16]. Therefore, it is believed that the higher power density formation in the P3-MFC compared to the others is due to the higher amount of poplar shavings. The decrease in current value and power density value may be due to the accumulation of inert metabolic products due to microbial activities.

### 3.2. Fourier Transform Infrared Spectroscopy Analysis of Poplar Wood Shavings and Electrodes

**Figure 4:**
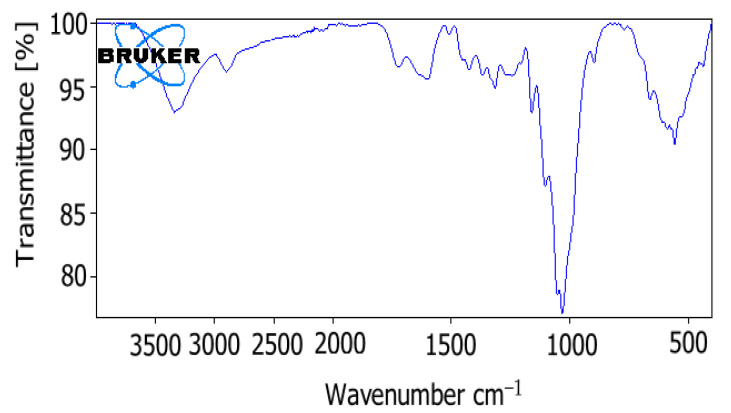
FTIR analysis of poplar wood.

Fourier transform infrared spectroscopy (FTIR) is one of the commonly used methods to detect and measure the chemical composition of wood material [17]. It is a biopolymer material because it contains biopolymer materials such as cellulose, hemicellulose, and lignin in the structure of poplar wood shavings [2,17,18]. The wood cell wall is mainly composed of cellulose, hemicellulose, and lignin. Because of all these chemical components, O–H stretching absorption bands (around 3400 cm^−1^) and C–H absorption bands (around 2927 cm^−1^) are usually formed [17]. In a study examining lignin properties in poplar wood chips, while 1597, 1509, and 1419 cm^−1^ bands in the fingerprint area were associated with aromatic skeletal vibrations, the C–H stretching vibration of the aromatic ring appeared at 1462 cm^−1^. The presence of peaks at 1326, 1268, and 1126 cm^−1^ (typical aromatic C–H in-plane deformation vibrations) indicated the features of various lignin types [18]. In a study examining the FTIR analysis of poplar, strong O–H stretching and C–H stretching absorptions were observed at 3367 and 2914 cm^−1^ due to the hydroxy groups and many C–H bonds in their structures due to cellulose, hemicellulose, and lignin. Absorption at 1745 cm^−1^ was due to the C=O stretch in hemicellulose and lignin. Absorption at 1618 cm^−1^ was attributed to the asymmetric tensile band of the glucuronic acid carboxyl group in hemicellulose and to the C=O stretch in the conjugated carbonyl of lignin. The tape at 1650 cm^−1^ was thought to possibly originate from adsorbed water. Higher absorption was also observed at 3367 cm^−1^ due to the moisture content in poplar biomass. At 1424 cm^−1^, bands were observed due to symmetrical CH_2_ bending vibration in cellulose, carboxyl vibration in xylan and glucuronic acid, and C–H in planar deformation with aromatic ring stress in lignin [19]. FTIR analysis of poplar tree used as the substrate for MFCs in this study is given in Figure 4. Here, the prominent FTIR regions of poplar wood shavings were approximately 900, 1100, 1250, 1330, 1420, 1510, 1610, 1750, 2920, and 3400 cm^−1^. FTIR analysis confirmed that poplar wood shavings contain cellulose, hemicellulose, and lignin materials. FTIR spectrum of the poplar wood shavings used in our study was similar and compatible with the study conducted by Zhang et al. (2020) [19].

**Figure 5:**
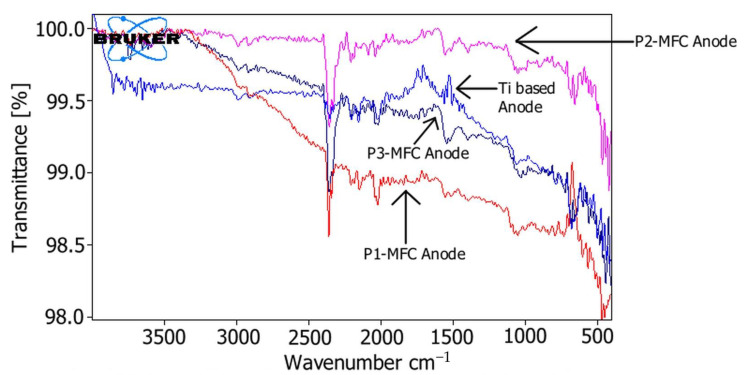
FTIR analyses of Ti-based anodes of MFCs.

As stated in previous studies [2], a biofilm layer forms on the anode electrode surface due to the reactions of microorganisms with the poplar tree used as a substrate. In order to understand the chemical composition relationship between this biofilm structure and the poplar wood structure, we performed FTIR analysis of the anode electrodes of MFCs at the end of the experiments. The FTIR spectrum of the Ti-based anode electrode at the beginning of the experiments and the FTIR spectrum of the anode electrodes used in MFCs are shown in Figure 5. According to the FTIR spectrum of the Ti-based anode at the beginning of the experiments, we observed waves around the 600 cm^−1^ region, 1000 cm^−1^ region, 2000 to 2400 cm^−1^ region, and 2900 to 3000 cm^−1^ region. It was believed that these wave regions were due to the interaction of titanium and oxygen atoms. However, Ti anode FTIR analysis showed bands corresponding to Ti–O–Ti stress due to TiO_2_ forming the Ti anode structure, and centering peaks belonged to the O–Ti–O bond in TiO_2_ [20]. In the FTIR spectrum of the anode used in P1-MFC, the approximate 500 cm^−1^ regions, 732 cm^−1^ regions, and regions between 1500 and 2400 cm^−1^ attracted attention. In the FTIR spectrum of the anode used in P2-MFC, it was the approximate between 419 and 435 cm^−1^ regions, 656 cm^−1^ regions, and regions between 1500 and 2500 cm^−1^ that attracted attention. In the FTIR spectrum of the anode used in P3-MFC, the approximate 442 cm^−1^ regions, 1990 cm^−1^ regions, and regions between 1500 and 2500 cm^−1^ attracted attention.

### 3.3. Scanning Electron Microscopy and Energy-Dispersive Spectrometer Analysis

Scanning electron microscopy (SEM) images were examined to understand the surface morphology of the anode electrodes, and energy-dispersive spectrometer (EDS) data were analyzed to understand element changes. EDS data are given in Table 1. According to EDS data, carbon was not seen in the Ti-based anode structure at the beginning of the experiments. At the end of the experiments, the carbon ratios of the Ti-based anode used in P1-MFC, P2-MFC, and P3-MFC were determined as 6.38, 11.89, and 14.53, respectively. When the oxygen ratios of Ti-based anodes of P1-MFC, P2-MFC, and P3-MFC were compared, the oxygen rate in P3-MFC was the highest. Due to the increase in the proportion of poplar wood shavings in MFCs, the carbon and oxygen ratios of Ti-based anode electrodes also increased. As a result, the highest carbon and oxygen ratios were seen in P3-MFC’s anode electrode.

**Figure 6:**
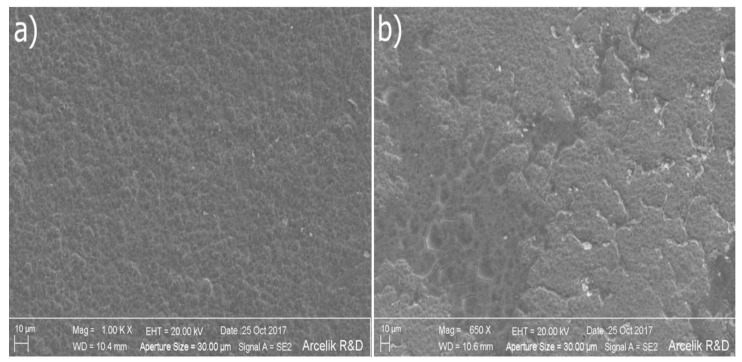
SEM images of Ti-based anodes: (**a**) Ti-based anode at the beginning of the experiments; (**b**) Ti-based anode at the final of experiments.

Morphological changes were present in SEM images of anodes at the P1-MFC, P2-MFC, and P3-MFC. However, since the most striking morphological change is in the P3-MFC anode, the SEM image of the P3-MFC anode is given in Figure 6. Looking at the SEM images, we saw that at the beginning of the experiments, Ti-based anode had an uncracked, homogeneous, and compact structure (Figure 6a). It was observed that a film layer and many cracks were formed on the Ti-based anode surface used in MFCs, and therefore a non-homogeneous structure with mixed surface morphology was formed (Figure 6b).

### 3.4. Bacteria Analyses and Biofilm

**Figure 7:**
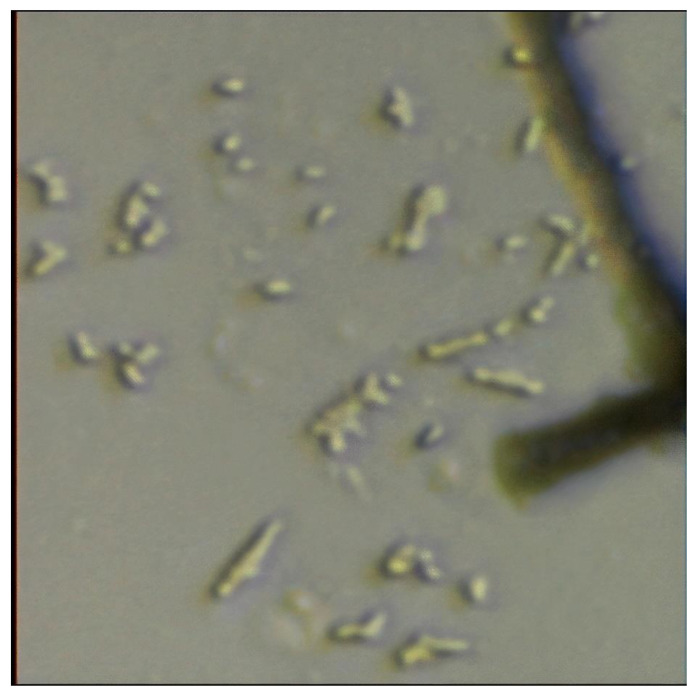
Microscope view (40×) of bacteria and poplar shavings.

Soil microbial structure was studied using an optical microscope at the beginning of the MFC experiments. In addition, electrolytes of MFCs consisting of soil, poplar wood shavings, and water were examined with the optical microscope at the end of the experiments. Optical microscope image of bacteria and poplar wood shavings is given in Figure 7. *Bacillus* species and *Coccus* species were observed in all-optical microscopic examinations in this study. The detection of these species is consistent with previous work on poplar-based MFC [2]. However, poplar wood shavings contained in the electrolyte of the MFCs were also viewed under the microscope. The organic material of the poplar wood structure is decomposed by *Bacillus* species and *Coccus* species bacteria. As a result, protons (hydrogen ions) and electrons are released. Here, the process of transferring electrons to the electrons is important, because transferring electrons directly to the electrons is a great advantage. For example, it has been suggested that the microorganism with its own electron shuttle is advantageous because it can be located at a certain distance from the electrode and transfer electrons to the electrode surface [21]. It is considered that the Bacillus species and Coccus species in this study create a suitable habitat for them in the electrolyte of poplar-based microbial fuel cells and have the ability to transfer electrons directly to the anode electrodes. Therefore, because of all these abilities of these bacteria, their existence is advantageous.

Since bacteria decompose the poplar tree, it is believed that the organic polymer materials that make up the structure of the poplar tree dissolve and form a film layer on the Ti-based anode electrode. Because when we look at FTIR analyzes, we see that due to polymer materials such as lignin, cellulose, and hemicellulose that make up the poplar tree, the FTIR analyses of Ti-based anode electrodes and poplar wood are close. In addition, SEM images and EDS analysis confirmed the formation of a film layer on the titanium-based anode electrode.

Bacteria play an active role in the decomposition of poplar woods and FTIR analysis of the layer on the surface of Ti-based anode electrodes of MFCs and FTIR analysis of polymers structures in poplar are similar. Therefore, it may be this film layer formed on the Ti-based anode electrode surface is a biofilm layer, also called extracellular polymeric materials. In addition, in the EDS analysis of the anodes of MFCs, as the ratio of poplar wood shavings in MFC increases, the ratio of carbon and oxygen increases, also supporting this idea.

## 4. Discussion

**Figure 8:**
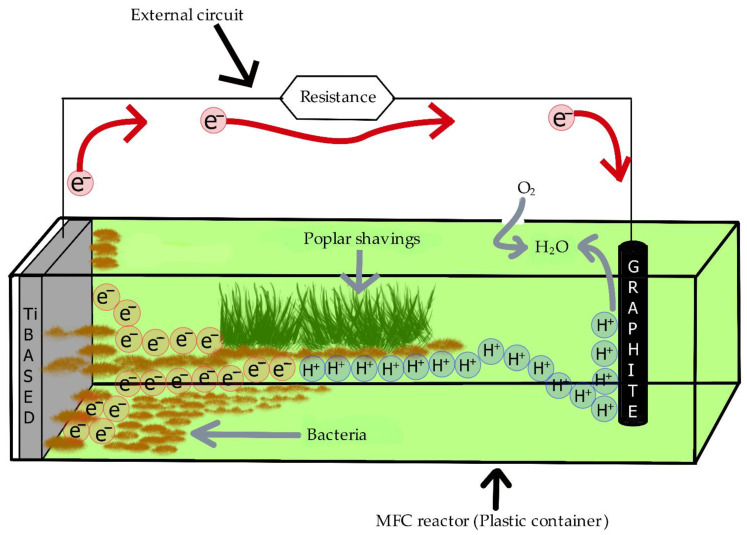
Working principle of poplar substrate-based MFCs.

The natural soil used in this study was a suitable habitat for *Bacillus* and *Coccus* species of bacteria. Therefore, in this MFC study, soil was used as a bacterial habitat, and poplar wood shavings were used as a substrate due to their rich organic material content. Titanium (Ti)-based material was used as anode electrode, and graphite was used as cathode electrode. After the electrodes were placed in the MFC and water was added, biochemical reactions started. Since poplar wood shavings were used as organic material, bacteria met their own nutritional needs by breaking down the organic materials that make up the poplar wood shavings, and at the same time, electrons (e^−^) and protons (hydrogen ions (H^+^)) were released. Protons migrate through the electrolyte to the cathode electrode. Since bacteria form a biofilm layer in the Ti-based anode layer of MFCs, electrons reach the anode electrode with this biofilm. Following that, electrons move to the cathode electrode through the outer circuit and react with hydrogen ions and air (O_2_) to form water. Thus, MFCs with Ti-based anode containing poplar substrate generate electrical energy. The working principle of MFCs with Ti-based anode containing poplar substrate is shown in Figure 8. In this study, P3-MFC provided the highest power generation density with a value of 5.11 mW/m^2^. This power density value was higher than the highest value (3.16 mW/m^2^) produced by the compost-based microbial fuel cell using urea as fuel [12]. In addition, the highest power density value produced in this study was higher than the maximum power density (3 mW/m^2^) of the terrestrial microbial fuel cell study [14].

In the previous study, the electrical generation performance of MFCs using various substrates was correlated with the biofilm-forming bacterial activity on the electrode surface [1,2,3,4,5,6,7]. In this research, the biofilm-related findings are consistent with previous studies [1,2,5,6,7,8]. Biofilm structure affects the electrical performance of MFCs. As seen in the SEM images (Figure 6b), we found that the ohmic losses of MFCs were increased due to the cracks that occurred in the biofilm. The increase in ohmic losses resulted in a lower power density of the MFCs. However, this may be related to the polarization curves and the shape of the power curves, with the losses in them being highly dependent on the activity of the *Bacillus* and *Coccus* species of bacteria.

In this study, it is believed that as a result of the decomposition of poplar wood shavings by bacteria, polymer materials such as cellulose, hemicellulose, and lignin in the structure of poplar tree dissolve, taking place in the biofilm structure formed on the surface of Ti-based electrodes. The FTIR analysis in this study was compared with the FTIR analysis of a previous study [19]. Looking at the FTIR analysis of the anode electrodes, we saw that the poplar tree was closely related to the FTIR analysis given in Table 2. In addition, it is understood that the increase in carbon and oxygen ratios in EDS analysis clearly showed that the organic materials contained in the poplar wood shavings covered the anode surfaces. Moreover, bacteria were seen around the plant fiber on optical microscope images (Figure 7). There was no noticeable deformation in the morphology of the bacteria. Therefore, it was shown that plant fiber and bacteria form a harmonious living space. As a result, FTIR, SEM, EDS, and optical microscope images confirmed the formation of a polymeric biofilm on the Ti-based anode electrode surface. It may be that one of the reasons why this biofilm has a polymer structure is that the polymer materials in the structure of the poplar tree adhere to the Ti-based anode electrode surface as a result of the catalysis reactions of bacteria. Moreover, bacteria secrete extracellular polymeric substances while forming a biofilm on the electrode [7,22]. Bacteria can produce extracellular polymeric materials during growth on the anode electrode surface. Extracellular polymeric materials can make up 50–90% of the total organic carbon content of biofilms. Extracellular polymeric materials can provide many benefits, such as ensuring the initial attachment of cells to solid surfaces, maturing the biofilm structure, and increasing biofilm resistance to environmental stresses [23]. 

This study can be applied to microbial fuel cells, and it is an inspiration for the development of low-power microbial fuel cells. This study, by drawing attention to the biofilm formed on the electrode surface as a result of the reaction of the poplar wood shavings substrate with bacteria, provided important information about the relations of the substrate, bacteria, and electrode with each other.

## 5. Conclusions

In the present study, biofilm formation and properties on the Ti-based anode electrode surface used in MFCs containing poplar substrate were characterized. Among MFCs containing different amounts of poplar wood shavings, the highest power density was achieved by the P3-MFC (5.11 mW/m^2^) with the highest amount of poplar wood shavings. The P3-MFC’s peak power densities were 3.54 times and 21.29 times higher, respectively, compared to P2-MFC and P1-MFC. On optical microscope images, the *Bacillus* and *Coccus* species of bacteria were seen around the fibers of poplar wood shavings. This confirmed that *Bacillus* and *Coccus* species of bacteria decompose organic matter found in poplar shavings. The transfer of electrons resulting from the breakdown of poplar wood shavings by bacteria took place through the biofilm on the Ti-based anode in the MFCs. At the end of the experiments, the biofilm layer formed on the Ti-based anode electrode surface. SEM, EDS, and FTIR analysis both confirmed the biofilm formation and showed that the biofilm was affected by the poplar structure used as a substrate. In other words, SEM, EDS, and FTIR analysis showed that the polymeric (cellulose, hemicellulose, lignin, etc.) materials in poplar wood shaving structure are transferred to the biofilm structure on the Ti-based anode electrode surface. Since bacteria and extracellular polymeric substances formed the biofilm structure, the biofilm was affected by the structure of the poplar substrate. Thus, thanks to the MFC application, the Ti-based anode electrode surface was coated with the polymer biofilm released from the poplar wood shavings as a result of the catalysis reactions of bacteria.

## Figures and Tables

**Table 1:** EDS data of MFC anodes.

Element (Weight %)	Ti-Based Anode	P1-MFC	P2-MFC	P3-MFC
Titanium	87.40	75.20	62.50	51.48
Oxygen	12.60	18.42	25.61	33.99
Carbon	0.0	6.38	11.89	14.53

**Table 2:** FTIR band assignments of poplar and its major components: cellulose, hemicellulose, and lignin [19].

Wavenumber (cm^−1^)	Assignment	Components
3367	O–H stretching	cellulose, hemicellulose, lignin
2914	C–H stretching	cellulose, hemicellulose, lignin
1745	C=O stretching	hemicellulose, lignin
1618	Aromatic skeletal vibration, C=O stretching, adsorbed O–H	hemicellulose, lignin
1508	C=C–C aromatic ring stretching and vibration	lignin
1457	C–H deformation (in methyl and methylene)	lignin
1424	Symmetric CH_2_ bending vibration, symmetric stretching band of carboxyl group, C–H deformation	cellulose, hemicellulose, lignin
1370	C–H bending, C–H stretching in CH_3_	cellulose, hemicellulose, lignin
1317	CH_2_ wagging, C–O stretching of C_5_ substituted aromatic units	cellulose, hemicellulose, lignin
1235	C–O stretching of guaiacyl unit	lignin
1160	C–O–C stretching	cellulose, hemicellulose
1108	Aromatic C–H in plane deformation	lignin
1053	C–OH stretching vibration, C–O deformation	cellulose, hemicellulose, lignin
1032	C–O stretching, aromatic C–H in plane deformation	cellulose, lignin
896	C–O–C stretching	cellulose, hemicellulose
846	Aromatic C–H out of plane bending	lignin

## Data Availability

The data presented in this study are available on request from the corresponding author.
